# Demographic processes limit upward altitudinal range expansion in a threatened tropical palm

**DOI:** 10.1002/ece3.4686

**Published:** 2018-11-26

**Authors:** Aline C. de Souza, Rita C. Q. Portela, Eduardo A. de Mattos

**Affiliations:** ^1^ Departamento de Ecologia, Instituto de Biologia Universidade Federal do Rio de Janeiro (UFRJ) Rio de Janeiro Brazil

**Keywords:** altitudinal gradient, climate change, *Euterpe edulis*, life table response experiment (LTRE), plant population and community dynamics, population growth rate, range limits, vital rates

## Abstract

Understanding the factors that determine species’ range limits is a key issue in ecology, and is fundamental for biodiversity conservation under widespread global environmental change. Elucidating how altitudinal variation affects demographic processes may provide important clues for understanding the factors limiting current and future species distributions, yet population dynamics at range limits are still poorly understood. Here, we tested the hypothesis that lower abundance at a species’ upper altitudinal range limit is related to lower vital rates. We compared the dynamics of two populations of the tropical palm *Euterpe edulis*, located near and at the edge of its altitudinal limit of distribution in the Brazilian Atlantic Forest. Data from four annual censuses, from 2012 to 2015, were used. We used matrix population models to estimate asymptotic population growth rates and the elasticity values for the vital rates of the two populations of *E. edulis*. Life table response experiments were used to compare population performance by measuring the contribution of each vital rate to population growth rates. Population growth rates were not significantly different from one in either population, indicating that both populations were stable during the study period. However, the abundance of all ontogenetic stages was lower at the altitudinal range limit, which was related to decreases in some vital rates, especially fecundity. Additionally, there were higher elasticity values for the survival of immatures and reproductive individuals, compared to all other vital rates, in both populations. *Synthesis*. Our results show that even a small‐scale environmental variation near range limits is sufficient to drive changes in the demography of this threatened palm. A minor increase in elevation approaching the limit of altitudinal distribution may reduce environmental suitability and affect population vital rates, thus contributing to setting upper altitudinal range limits for plants.

## 1 INTRODUCTION

A central goal in ecology and evolution is to explain the causes of species’ range limits (Geber, 2008; Sexton, McIntyre, Angert, & Rice, 2009). One hypothesis for why some species stop occurring at an apparently arbitrary point along a continuous environmental gradient is the presence of unsuitable abiotic and biotic conditions at this range edge (Brown, Stevens, & Kaufman, 1996). In other words, the species’ range limit coincides with the limit of its ecological niche, and the species is maladapted to the environmental conditions beyond this limit (Hargreaves, Samis, & Eckert, 2014; Sexton et al., 2009). According to this hypothesis, at their range edge, populations exhibit reduced performance, size, and genetic diversity, which increase their vulnerability to extreme events. This would ensure that populations are not self‐sustaining beyond the range edge (Abeli, Gentili, Mondoni, Orsenigo, & Rossi, 2014; Gaston, 2009; Hargreaves et al., 2014).

The study of population dynamics is an essential part of investigating whether marginal populations coincide with the limits of a species’ ecological niche, resulting in a decrease in demographic parameters (Gaston, 2009). Some studies have observed decreases in demographic parameters, such as abundance, fecundity, and survival, at range limits (Eckhart et al., 2011; Jump & Woodward, 2003; Marcora, Hensen, Renison, Seltmann, & Wesche, 2008; Vaupel & Matthies, 2012). However, other studies have not observed a consistent pattern of lower performance or abundance at the range edge (Abeli et al., 2014; García, Goñi, & Guzmán, 2010; Villellas, Ehrlén, Olesen, Braza, & García, 2013; Wagner et al., 2011), showing that geographical and ecological margins are not necessarily coincident (Soulé, 1973). Dispersal barriers may limit the distribution of these species where local environmental conditions beyond the range edge are still suitable (Primack & Miao, 1992). Another possible explanation is demographic compensation under unfavorable conditions at the range edge, whereby reductions in some vital rates are compensated for by increases in others, resulting in similar population growth rates (Doak & Morris, 2010; Villellas, Doak, García, & Morris, 2015).

The study of marginal populations has increased recently due to recognition of the potential impacts of climate change, habitat loss and fragmentation, and invasive species on species range (Grayson & Johnson, 2017; Hampe & Petit, 2005; Rehm, Olivas, Stroud, & Feeley, 2015). Marginal populations are natural laboratories in which to study the limits of adaptation or occurrences of unique local adaptations. They could also be important for conservation, as they may hold important genetic variations which may even result in distinct ecotypes (Kawecki, 2008; Lesica & Allendorf, 1995). In addition, the study of population dynamics at species’ range limits could be useful for predicting both the effects of climate change on the probability of local persistence and whether species will contract or expand their distribution (Hampe & Petit, 2005; Normand, Zimmermann, Schurr, & Lischke, 2014).

The range limits of many species are frequently linked to changes in environmental conditions caused by abrupt habitat transitions, such as at the boundary between terrestrial and aquatic environments. However, in many species, range limits occur in areas without such clear changes in environmental conditions (Hargreaves et al., 2014; Sexton et al., 2009). Across an altitudinal gradient, small increases in altitude can drive significant alterations in abiotic conditions, such as decreases in temperature and total atmospheric pressure (Körner, 2007). Thus, populations separated by relatively short distances along an altitudinal gradient can be exposed to different selective pressures (Körner, 2007). These different environmental conditions and selective pressures may drive differences in population dynamics, including changes in survival, growth, and fecundity (Angert, 2009; García‐Camacho, Albert, & Escudero, 2012; Giménez‐Benavides, Albert, Iriondo, & Escudero, 2011; Pollnac, Maxwell, Taper, & Rew, 2014).

However, little is known about population dynamics in plants across altitudinal gradients, especially in tropical areas. Analyses of population dynamics along tropical mountain ranges are necessary for understanding the demographic processes affecting distribution and abundance and for predicting species’ responses to climate change in such high‐diversity ecosystems (Giménez‐Benavides et al., 2011). For example, it is well known that climate change may cause altitudinal range shifts in some species, including non‐native species, and have negative impacts on local communities (Seipel, Alexander, Edwards, & Kueffer, 2016; Wilson et al., 2005).

Here, we analyzed the population dynamics of a tropical palm, *Euterpe edulis*, along a very short altitudinal gradient**.** We tested the hypothesis that decreases in the abundance of this palm at the upper altitudinal limit were associated with decreases in demographic parameters. The upper altitudinal limit of *E. edulis* has been suggested to be around 1,000 m (Henderson, Galeano, & Bernal, 1995), although the causal factors underlying this limit are not yet clear. Specifically, we addressed the following questions. (a) Does small‐scale variation in environmental conditions associated with elevation near the upper altitudinal limit drive significant decreases in vital rates? (b) Does the contribution of each vital rate to population growth vary along the altitudinal gradient? (c) Is the population at the upper altitudinal limit contracting, expanding or stable? The answers to these questions will advance our understanding of the causes of range limits along altitudinal gradients for tropical species and may be useful for predicting the effects of global warming on species’ altitudinal ranges.

## 2 MATERIAL AND METHODS

### 2.1 Study species

The palm *Euterpe edulis* Mart. has a wide distribution range, occurring in the Brazilian Atlantic Forest from sea level to around 1,000 m a.s.l., in the gallery forests of Cerrado in Brazil, and also extending into Argentina and Paraguay (Henderson et al., 1995). It is a shade‐tolerant, monoecious tree with a solitary stem, and is a dominant tree in pristine forest areas (Henderson et al., 1995; Silva‐Matos, Freckleton, & Watkinson, 1999). The flowers are produced annually and are pollinated by beetles, bees, and the wind (Mantovani & Morellato, 2000). The palm's single‐seeded fruits are produced annually and are consumed by a large variety of animals**;** they are considered to be a keystone resource in the Atlantic Forest (Galetti, Zipparro, & Morellato, 1999). The species forms a transient seed bank but an expressive seedling bank, with around 1,200 seedlings/ha at some sites (Reis et al., 2000). Many local populations of the palm have been reduced or gone extinct due to overexploitation of its palm heart, and large populations are now restricted to protected areas (Silva‐Matos et al., 1999).

### 2.2 Study system

The study area was in the Serra dos Órgãos National Park (PARNASO; 22°23′S 43°10′W), Rio de Janeiro, Brazil. The park encompasses ca. 20,000 ha and is located in one of the largest contiguous remnants of the Brazilian Atlantic Forest (Ribeiro, Metzger, Martensen, Ponzoni, & Hirota, 2009). The vegetation is predominantly characterized by Montane Ombrophilous Dense Forest (Veloso, Rangel‐Filho, & Lima, 1991). The area has a Cfb climate, according to the Köppen classification system, that is, it is mesothermic. The mean annual precipitation recorded by weather stations in Serra dos Órgãos National Park is around 2,000 mm. Precipitation is highest during the summer, is characterized by a superhumid period from October to March, and is lower from June to August. However, true dry periods are rare, since there is generally high precipitation throughout the year, frequent mist, and mild temperatures (even in the summer) due to the elevation (Nimer, 1989). All of these factors act synergistically to result in the likely absence of water stress, a distinctive characteristic of this area (Castro, 2008).

In the study area, *E. edulis* is the most abundant species in the arboreal community (R. Finotti, unpublished data). A decline in the density of *E. edulis* at the study site is clearly evident when approaching its upper altitudinal limit of 1,400 m a.s.l. (A. C. Souza, pers. obs.). We selected two sites with different densities of *E. edulis*: one at the species’ altitudinal limit (around 1,400 m a.s.l.), with a lower density, and the other at a lower altitude (around 1,200 m a.s.l.), with a higher density. We assumed that the local environmental conditions at the lower‐altitude site were optimal for *E. edulis*, since the population density we recorded was the highest ever documented for this species (54,320 ind/ha), when compared to previous studies (Melito, Faria, Amorim, & Cazetta, 2014).

Air temperature and relative humidity (%) at each altitude were monitored with HOBO U23‐002 loggers (Onset Computer Inc., MA, USA), at 30‐min intervals from January 2013 to June 2014. For each climatic variable, we first recorded the lowest, the mean and the highest values for each month. Then, we calculated the mean values across the 18 months (Table 1). We also measured the frequency of occurrence of temperatures below 10°C at each altitude. Temperatures were on average 1.3°C lower at the higher‐altitude than at the lower‐altitude site (Table 1). In addition, the frequency of occurrence of very low temperatures (<10°C) was three times higher at the higher‐altitude than at the lower‐altitude site. The relative humidity (%) was similar among altitudes considering the average and maximum values, but minimum values were relatively lower at the higher‐altitude site (Table 1).

**Table 1:** Mean (±) of minimum, maximum and average temperature, and relative humidity (%) at each altitude from January 2013 to June 2014

Altitude	Temperature (°C)			RH (%)		
	Minimum	Maximum	Average	Minimum	Maximum	Average
Lower	10.04 ± 3.03	22.55 ± 2.68	16.13 ± 2.19	72.06 ± 10.29	100 ± 0	97.34 ± 2.51
Higher	8.87 ± 3.19	21.03 ± 2.56	14.94 ± 2.08	46.44 ± 19.31	100 ± 0	93.28 ± 4.00

### 2.3 Data collection

Data were collected during three annual transition intervals from 2012 to 2015. In 2012, all *E. edulis* individuals were marked with numbered tags and classified according to the four ontogenetic stages described below (adapted from Portela & Santos, 2011). We surveyed the two populations of *E. edulis* within randomly located plots, which differed in size and number at each site due to the different densities of *E. edulis* and microhabitat heterogeneity. This sampling design allowed us to analyze demographic transitions in areas large enough to capture the environmental variation at each site. At higher altitudes (1,300–1,395 m a.s.l.), plot sizes ranged from 2.5 to 300 m^2^, with a total sampled area of 0.21 ha. At lower altitudes (1,175–1,235 m a.s.l.), plot sizes ranged from 2.0 to 300 m^2^, with a total sampled area of 0.15 ha. In addition, the total area sampled for *E. edulis* seedlings and saplings was smaller at each site, since the density of these ontogenetic stages was much higher than that of the immature and adult stages.

Annual censuses were conducted for all ontogenetic stages except seedlings from 2012 to 2015 during the winter (July, August, and September), to measure annual survival, growth, retrogression, reproductive status (inflorescence or fruit production), and fecundity. Data collection for seedlings occurred twice a year, once during the wet season and once during the dry season, to evaluate potential differences in seedling mortality and recruitment between seasons. At each census, we measured the survival and growth of all seedlings and tagged new seedling recruits. Plant fecundity was estimated annually as the ratio of the number of new seedlings at time *t + *1 to the number of reproductive individuals at time *t* at each site. The total number of *E. edulis* individuals of each ontogenetic stage was recorded for all plots to estimate the plant density of each stage.

### 2.4 Stage classification

At each site, we classified *E. edulis* individuals into one of the following four ontogenetic stages based on morphological analysis (adapted from Portela & Santos, 2011). Seedlings were defined as stemless individuals with palmate leaves. Saplings were defined as stemless individuals with pinnate and occasionally palmate leaves. Immatures were defined as individuals with stems but no reproductive structures. Finally, reproductive individuals were defined as those with stems and flowers and/or fruit.

### 2.5 Matrix analysis

For each site, we built three Lefkovitch matrices using demographic parameters obtained from the data collected during each annual transition interval (Caswell, 2001). Each projection matrix can be described by the equation: *n*(t + 1) = A*n(t), where ***n*(t)** and ***n*(t + 1)** are vectors representing the abundance of different stages at times *t* and *t + *1, and **A** is the projection matrix. The projection matrix is composed of matrix elements (*a_ij_*) that represent the transition probabilities or fecundity rates, describing how stage *j* at time *t* contributes to stage *i* at time *t + *1 (Caswell, 2001). Thus, the matrix elements (or upper‐level vital rates) represent stasis (*S_ij_*), retrogression (*R_ij_*), growth (*G_ij_*), and fecundity (*F_ij_*). Therefore, each matrix element is a function of lower‐level vital rates (survival, growth, retrogression, and reproduction).

**Figure 1:**
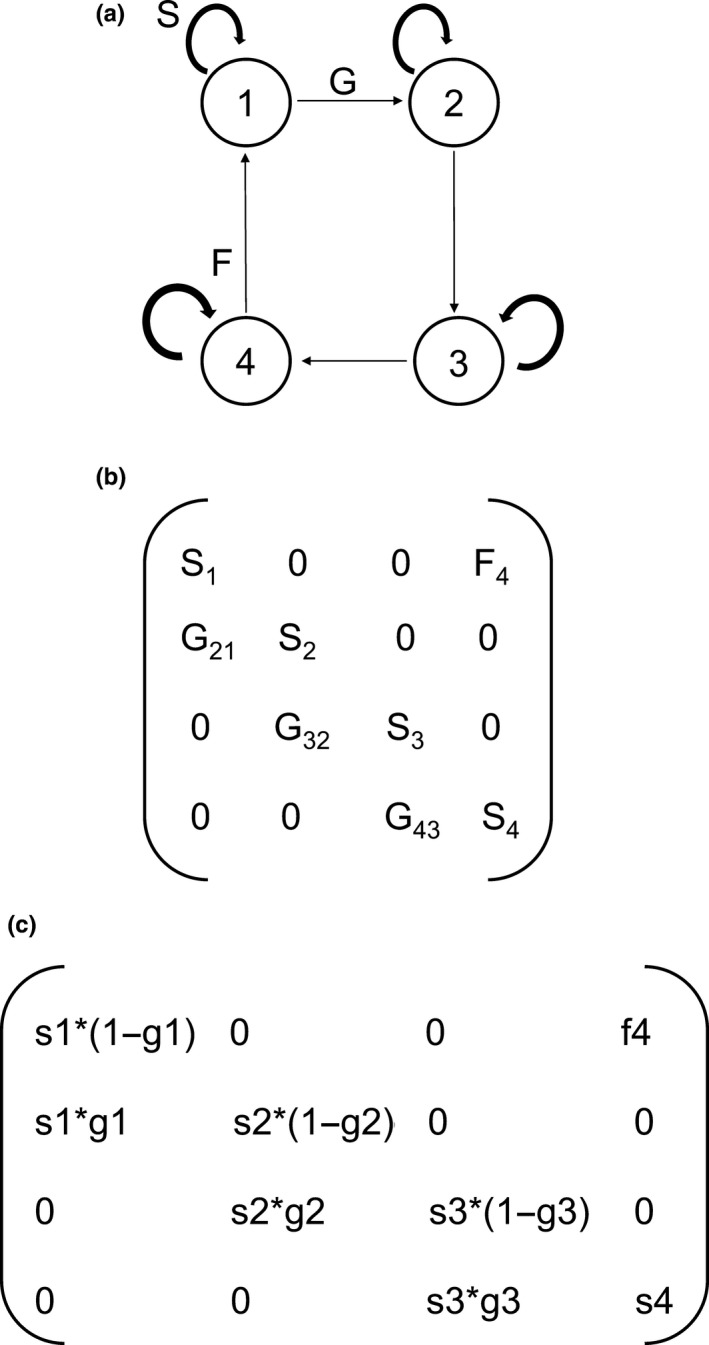
(a) Life cycle graph for *Euterpe edulis*. Circles represent the stages and arrows represent potential transitions between the stages. (b) Population projection matrix corresponding to the life cycle graph in (a). The matrix elements represent the probability of survival and remaining in the same stage (*S_ij_*), the probability of survival and growth to the next stage (*G_ij_*), and the mean fertility per reproductive individual (*F_ij_*). In this model, we treated all transition intervals as annual. (c) Population projection matrix corresponding to the matrix elements composed of the product of the lower‐level vital rates based on the life cycle graph in (a). *s1* seedling survival; *s2* sapling survival; *s3* immature survival; *s4* reproductive survival; *g1* growth of seedling to the sapling stage; *g2* growth of sapling to the immature stage; *g3* growth of immature to the reproductive stage; *f4* fecundity, as the ratio of the number of new seedlings at time *t* + 1 to the number of reproductive individuals at time *t*. Zero entries correspond to transitions that were not observed during the study

We constructed a projection matrix derived from the life cycle of *E. edulis* (Figure 1a), including all of the possible transitions between ontogenetic stages observed in the study model, which resulted in a 4 × 4 matrix (Figure 1b). For all matrix analyses, we used the “popbio” package (Stubben, Milligan, & Nantel, 2013) in R (R Development Core Team, 2014). We calculated the asymptotic population growth rate (*λ*; dominant eigenvalue) using each transition matrix (Caswell, 2001). To verify differences in *λ* between sites, we calculated bias‐corrected 95% confidence intervals (CIs) for each *λ* by bootstrapping. We constructed 2000 bootstrapped matrices by randomly sampling individuals, with replacements from the data for each stage, maintaining the same number of observations at each time interval. Then, the *λ* values of the 2,000 replications were averaged and 95% CIs were calculated using the percentiles of the distribution (Caswell, 2001).

We used prospective analyses to explore how proportional (elasticity) changes in matrix elements affected the population growth rate (*λ*; Caswell, 2001) using the “eigen.analysis” function in the popbio package (Stubben et al., 2013). To analyze the contribution of each vital rate to *λ*, we also calculated the elasticity of the lower‐level vital rates (underlying vital rates), since the population projection matrix elements are a function of more than one vital rate (Figure 1c; Caswell, 2001; Franco & Silvertown, 2004). The elasticity of lower‐level vital rates (survival, growth, retrogression, and fecundity) is more appropriate than the elasticity of the matrix elements for comparisons between populations, because the matrix elements are the products of the vital rates (Franco & Silvertown, 2004). To calculate the elasticity of each lower‐level vital rate, we used the “vitalsens” function in the popbio package.

### 2.6 Life table response experiment

A life table response experiment (LTRE) analysis was used to verify the contribution of each vital rate to the population growth rate (*λ*). We used a fixed‐design experiment, with the higher‐density population of *E. edulis* as a reference (Caswell, 2001):

$$\Delta \lambda =\lambda^{t}-\;\lambda^{c}\approx \sum_{\mathrm{ij}}\left(a_{ij\;-\;}^{t}a_{\mathrm{ij}}^{c}\right)\times \left.\left(\frac{\partial \lambda }{\partial a_{\mathrm{ij}}}\right)\right|\left(A^{i}+A^{c}\right)/2$$

where $\left(a_{\mathrm{ij}}^{t}-a_{\mathrm{ij}}^{c}\right)$ is the difference in *a_ij_* between the “treatment” matrix and the “control” matrix, and ∂*λ*/∂*a_ij_* is the sensitivity of *λ* to changes in *a_ij_* calculated for the mean matrix (or the matrix “midway”) between the treatment matrix and control matrix (Caswell, 2001).

### 2.7 Density of all ontogenetic stages

To analyze the effects of altitude (lower vs. higher altitude) on the density of each ontogenetic stage, we used a generalized linear model (GLM) with quasi‐Poisson error distribution and log‐link function (Crawley, 2013).

### 2.8 Seedling recruitment and mortality

To analyze the effects of altitude and season on seedling recruitment and mortality, we also used a GLM for each response variable (Crawley, 2013). For seedling recruitment, we used a negative binomial error distribution with a log‐link function. Since the total area sampled for seedling recruitment differed between altitudes, we calculated the number of new seedlings per 10 m^2^ for both altitudes. For seedling mortality (proportion of dead seedlings), we used a binomial error distribution with a logit link function (Crawley, 2013).

## 3 RESULTS

### 3.1 Asymptotic population growth rates (λ)

**Figure 2:**
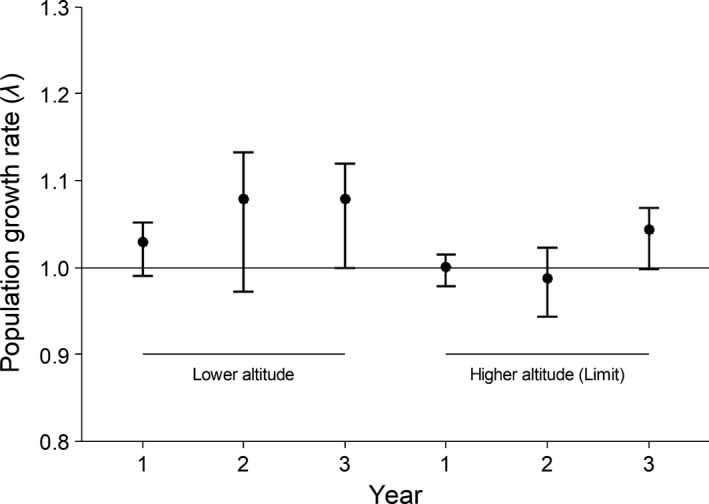
Asymptotic population growth rates (*λ*) for two populations of *Euterpe edulis* at lower (1,200 m) and higher (1,400 m) altitudes. Each point represents the mean, and the error bars represent the 95% CIs after bootstrapping each annual transition matrix. Year codes: 1 = 2012–2013; 2 = 2013–2014; 3 = 2014–2015

The average asymptotic population growth rates of *E. edulis* varied from 1.02 to 1.07 at the lower‐altitude site and from 0.98 to 1.04 at the higher‐altitude site, across three annual transition intervals (Figure 2). Over the total study period, both populations were in a stable equilibrium, since their growth rates did not differ significantly from unity (*λ* = 1). In addition, there were no consistent differences between populations, since the CIs overlapped throughout the study period (Figure 2).

### 3.2 Elasticity analysis

**Figure 3:**
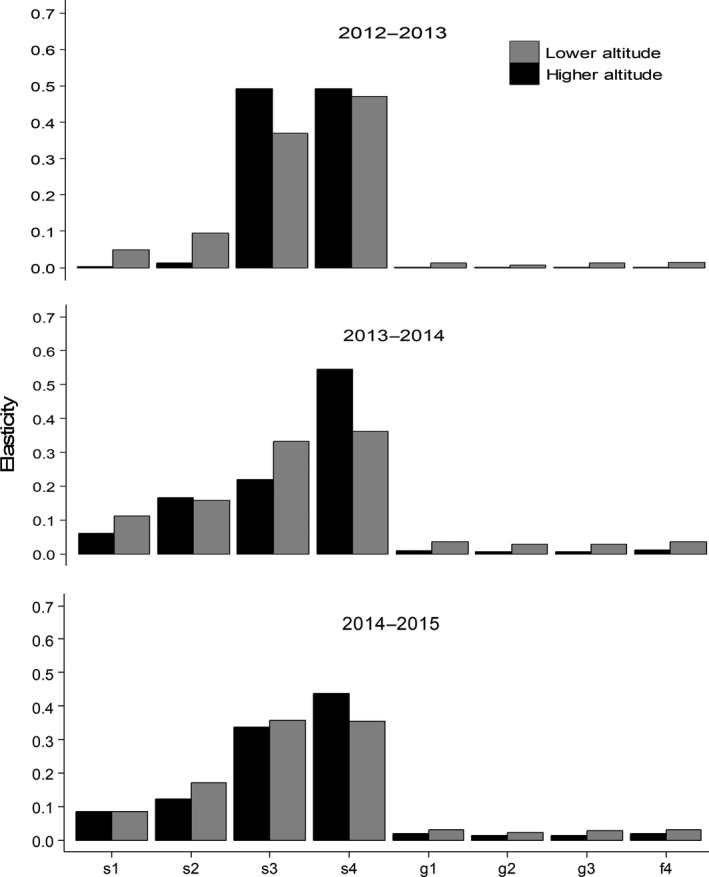
Elasticity of lower‐level vital rates for populations of *Euterpe edulis* at lower and higher altitudes. f: fecundity; g: growth; s: survival. The stages are as follows: 1 = seedling; 2 = sapling; 3 = immature; 4 = reproductive

In general, the contributions of different vital rates to *λ* were similar between the two populations of *E. edulis* across the study period (Figure 3). The elasticity values were highest for the survival of immature and reproductive individuals of both populations across all the transition years, ranging from 22% to 54%. Seedling and sapling survival had intermediate elasticity values, ranging from 0.002% to 0.17%. In contrast, fecundity and the growth of all ontogenetic stages made the lowest contribution to *λ* for both populations (Figure 3).

### 3.3 Life table response experiments (LTRE)

**Figure 4:**
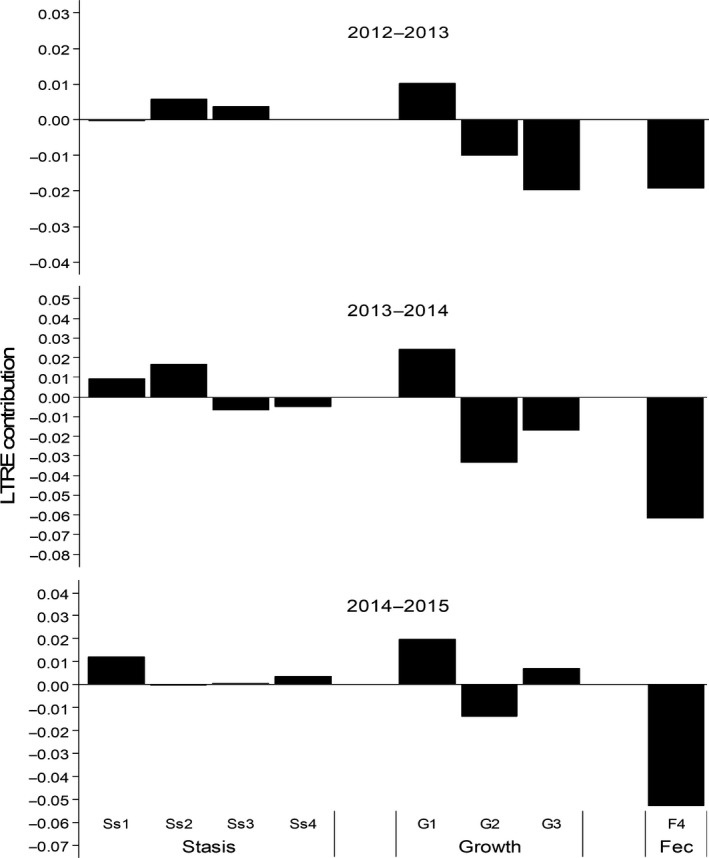
LTRE contributions of matrix elements representing stasis, growth, and fecundity in a population of *Euterpe edulis* located at a higher altitude compared with one at a lower altitude. One LTRE was performed for each transition year. F: fecundity; G: growth; Ss: stasis. The ontogenetic stages are as follows: 1 = seedling; 2 = sapling; 3 = immature; 4 = reproductive

The results of the LTRE analyses revealed that the contributions of each matrix element to *λ* differed between the two populations of *E. edulis*, despite the lack of a significant difference in population growth rates between them (Figure 4). At the higher‐altitude site, the contributions to the observed population growth rate of both fecundity (*F*) and the growth of saplings to immatures (*G*_2_) were consistently negative and high in magnitude. Thus, the population at the altitudinal range limit had lower values for these vital rates (fecundity and transition of saplings to immatures) than the control population (lower altitude). However, at the higher‐altitude site, the contribution to the observed population growth rate of the transition of seedlings to saplings (*G*_1_) was consistently positive, with an intermediate magnitude relative to the lower‐altitude population.

In the first transition year (2012–2013), we observed a larger negative contribution of the transition of immatures to reproductive individuals (*G*_3_) in the higher‐altitude population, and a negligible negative contribution of seedling stasis (*S*_1_; Figure 4). In the second transition year, except for seedling and sapling stasis, and the growth of seedlings to saplings, all other vital rates contributed negatively, resulting in a slight decrease in the population growth rate of the higher‐altitude population. On the other hand, in the last transition year, only fecundity and sapling growth contributed negatively.

### 3.4 Plant density

The density of all ontogenetic stages of *E. edulis* decreased significantly at the species’ altitudinal range limit (Table 2). In both populations, the seedling stage was the most abundant, especially at the lower‐altitude site, and the density of individuals decreased from the seedling stage to reproductive individuals (Table 2).

**Table 2:** Effects of altitude on density (no. individuals/m^2^) for different ontogenetic stages of *Euterpe edulis*

Ontogenetic stage	Lower‐altitude (1,200 m)	Higher‐altitude (1,400 m)	*p*
Seedling	4.791 ± 0.34	0.124 ± 0.08	<**0.001**
Sapling	0.557 ± 0.58	0.028 ± 0.01	<**0.001**
Immature	0.202 ± 0.03	0.023 ± 0.01	<**0.001**
Reproductive	0.034 ± 0.01	0.010 ± 0.01	**0.032**

Notes

For each ontogenetic stage, a generalized linear model was tested. Data are mean ± *SE* of each ontogenetic stage; values in bold represent a significant effect at *p* < 0.05, *df* = 1.

### 3.5 Seedling recruitment and mortality

**Figure 5:**
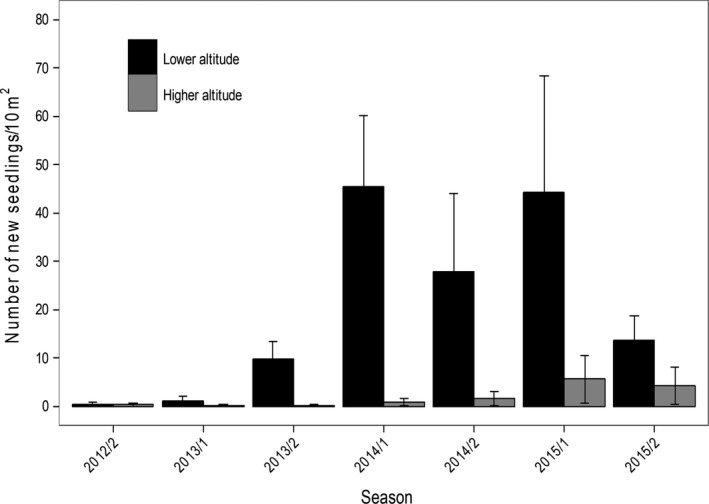
Seedling recruitment (mean ± *SE*) of *Euterpe edulis* at both altitudes during the wet season (1) and dry season (2) of different years of the study period. Seedling recruitment was calculated based on the initial number of seedlings present at each plot at the beginning of each season. To compare seedling recruitment between altitudes for which different amounts of area were sampled, we standardized the number of new seedlings to seedlings per 10 m^2^ for both altitudes

**Figure 6:**
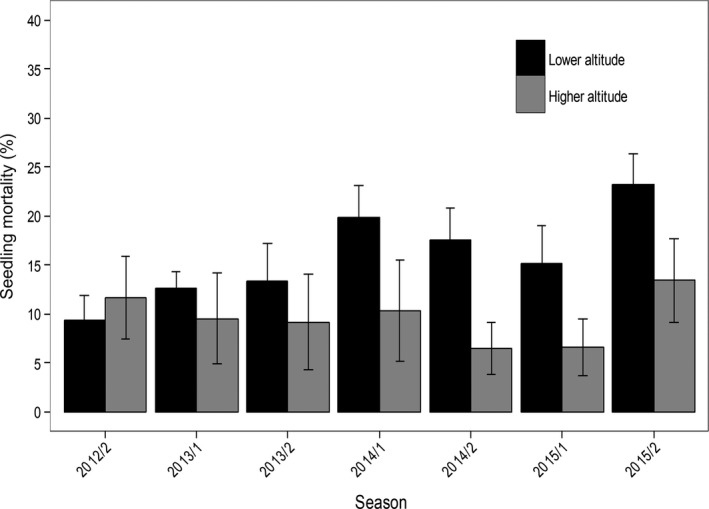
Seedling mortality (mean ± *SE*) of *Euterpe edulis* at lower and higher altitudes during the wet season (1) and dry season (2) of different years of the study period. Seedling mortality was calculated based on the initial number of seedlings present in each plot at the beginning of each season

Altitude had a significant effect on seedling recruitment and mortality, with higher values at lower altitude. On the other hand, neither season nor the interaction between altitude and season had significant effects on seedling recruitment and mortality (Table 3; Figure 5, and Figure 6).

**Table 3:** Effects of altitude, season, and their interaction on seedling recruitment and mortality in *Euterpe edulis*

Variable	Seedling recruitment		Seedling mortality	
	*z*‐value	*p*	*z*‐value	*p*
Altitude	3.179	**0.001**	2.876	**0.004**
Season	−0.466	0.641	1.655	0.098
Season × Altitude	−0.463	0.643	−1.057	0.290

For each response variable (seedling recruitment or mortality), a separate GLM was tested, with altitude, season, and their interaction as explanatory variables. The values in bold represent a significant effect at *p* < 0.05, *df* = 1.

## 4 DISCUSSION

Classical theory predicts that decreases in population abundance at range limits are related to lower performance, with reductions in vital rates such as fecundity, growth, and survival due to unsuitable environmental conditions (Brown, 1984). However, previous studies have shown conflicting results, illustrating that geographic range limits do not always coincide with ecological margins (Angert, 2009; Pironon et al., 2016; Sagarin & Gaines, 2002; Villellas, Ehrlén, et al., 2013). On a local scale, our results support the prediction that environmental suitability is lower at range limits, since we observed reductions in abundance and some vital rates, especially fecundity and growth. These findings show that even small‐scale environmental variation near range limits may drive significant changes in plant demography. In addition, our study evaluated small‐scale variation in elevation, with two populations separated by a short distance. Thus, a minor increase in elevation approaching the limit of the altitudinal distribution may reduce environmental suitability and affect population vital rates, contributing to setting upper altitudinal range limits for plants.

Both of the *E. edulis* populations studied were stable during the study period, since their population growth rates (*λ*) did not differ statistically from unity. Contrary to our results, some previous studies in mountainous zones have found population growth rates to be below one in peripheral populations (Angert, 2009; Giménez‐Benavides et al., 2011; Villellas, Cardós, & García, 2016; but see García‐Camacho et al., 2012). Angert (2009) suggested that marginal populations of *Mimulus lewisii* were demographic sinks that were maintained by immigration. Giménez‐benavides et al. (2011) and Villellas et al. (2016) also found reduced population growth rates at lower altitudinal limits and proposed that altitudinal range contraction is occurring in response to climate change. For *E. edulis*, growth rates close to one were observed in both of the sampled populations, providing evidence that this threatened tropical palm did not retract or expand its altitudinal range distribution during the study period. Thus, despite reductions in performance and abundance at the higher‐altitude site, this population does not represent a demographic sink population that is maintained by immigration, since birth and death rates were similar, as shown by the *λ* values across the study period.

Although both of the *E. edulis* populations we studied were stable, some vital rates were lower at the upper elevation limit. Similarly, Aikens and Roach (2014) studying a narrow endemic plant also found that in edge populations, a reduction in some vital rates was buffered by an increase in flowering probability, resulting in similar population growth rates in central and edge populations. In our study, the similar population growth rates found in the two populations of *E. edulis* may result from demographic compensation. This is because at the upper altitudinal limit, decreases in some vital rates, such as fecundity, were buffered by increases in other vital rates, as shown by the LTRE analysis. Therefore, demographic compensation may be important for edge populations of plant species in ensuring persistence under unsuitable environmental conditions. This contributes to species having larger ranges and occurring under a wider range of conditions, as shown by previous studies conducted on broader spatial scales (Doak & Morris, 2010; García‐Camacho et al., 2012; Villellas, Morris, & García, 2013). However, we cannot verify whether this demographic process operates across the small altitudinal range in our study, due to our sampling design. We recommend that future studies use the method proposed by Villellas et al. (2015) to evaluate whether demographic compensation is an important demographic process in *E. edulis*. This is an interesting direction for investigation because demographic compensation could contribute to the presence of this threatened palm under a range of environmental conditions along altitudinal gradients.

In our study, the LTRE analysis was important for revealing that although both populations show similar population growth rates, they had different dynamics; at higher altitude, fecundity was lower, but seedling survival and growth were higher. Reductions in seedling growth and survival at lower altitude could be related to the negative effects of density dependence, since at this site, the density of seedlings was much higher. It is important to assess density dependence in demographic studies, to be able to determine whether reductions in some demographic rates are associated with poorer habitat suitability or density‐dependent effects under higher population densities (Pironon et al., 2016). Indeed, the negative effect of density dependence could reduce performance in central populations with higher plant densities, counteracting the positive effects of greater habitat suitability (Kluth & Bruelheide, 2005b; Purves, 2009; Volis, Mendlinger, & Ward, 2004). Since negative effects of density dependence have already been observed in *E. edulis* seedlings (Silva‐Matos et al., 1999), we suggest that this regulatory mechanism is the main cause of the lower survival and growth of *E. edulis* seedlings at lower‐altitude site, rather than reduced habitat suitability.

The fecundity of *E. edulis*, measured as seedling recruitment per reproductive individual, as shown by the LTRE analysis, was lower at the species’ upper altitudinal limit (Figure 4). Previous studies have also observed a reduction in seedling recruitment at range limits (Tremblay, Bergeron, Lalonde, & Mauffette, 2002; Doak & Morris, 2010; Aikens & Roach, 2014; Lesica, 2014; but see Villellas, Ehrlén, et al., 2013). The poorer seedling recruitment of *E. edulis* at its upper altitudinal range limit could be associated with lower temperatures, because the success rate and speed of its germination are depressed at lower temperatures (Roberto & Habermann, 2010). In addition, the slow speed of transition from seeds into seedlings could increase the vulnerability of seeds and germinants to desiccation, since *E. edulis* seeds are recalcitrant (Andrade, 2001). Another factor that could constrain seedling recruitment is postdispersal seed predation, which is much higher at the upper altitudinal range limit (Souza et al. unpublished data). Some studies have also found reduced plant regeneration at the limit of distribution due to biotic factors, such as pre‐ and postdispersal seed predation and seedling herbivory. These findings provide evidence that abiotic factors are not the only ones that could constrain the ranges of plant species (Brown & Vellend, 2014; Bruelheide & Scheidel, 1999; Cairns & Moen, 2004; Jameson, Trant, & Hermanutz, 2015). Thus, decreases in seedling recruitment at the upper altitudinal range limit may represent an important demographic bottleneck that contributes to the low density of *E. edulis* at this location, highlighting the important role played by early life stages in setting distribution limits.

Another factor that may contribute to reductions in *E. edulis* density at its upper altitudinal range limit is the lower rates of transition from saplings to immatures and from immatures to reproductive individuals. The lower growth rates of these two ontogenetic stages act synergistically to delay the recruitment of the reproductive stage, resulting in later onset of reproduction at higher‐altitude site. In addition, the slower development of saplings and immatures increases the generation time, leaving the saplings and immatures of this species more vulnerable to environmental stochasticity, reducing their probability of reaching the reproductive stage (Jansen, Zuidema, Anten, & Martínez‐Ramos, 2012; Zuidema, Brienen, & During, 2009). Thus, since *E. edulis* individuals at lower altitudes grow faster (higher rates of transition to later ontogenetic stages), their fitness could be higher, since younger reproductive individuals can produce a larger total number of seeds, if we assume a similar risk of mortality in saplings and immatures, and similar annual reproductive output irrespective of altitude. Slower growth in saplings and immatures may be associated with poorer habitat suitability, for example, lower temperatures, at higher altitudes. Low temperatures have negative effects on photosynthesis and growth in *E. edulis* seedlings (Gatti, Campanello, Montti, & Goldstein, 2008) and could also reduce the growth rate of these ontogenetic stages (Brienen, Zuidema, & Martínez‐Ramos, 2010; Eguiarte, Pérez‐Naser, & Piñero, 1992).

The stasis of later ontogenetic stages (immature and reproductive individuals; Figure 3) was the demographic parameter with the greatest effect on *λ* in both *E. edulis* populations. This finding is in accordance with previous studies on long‐lived plant species such as palms (Franco & Silvertown, 2004; Kouassi, Barot, Gignoux, & Bi, 2008; Portela, Bruna, & Santos, 2010; Sampaio & Scariot, 2010). Similarly, the lower elasticity of sapling growth and fecundity explain why the negative contributions of these demographic processes did not significantly decrease the population growth rate at the altitudinal range limit. These results suggest that any factor inducing higher mortality in immature or reproductive individuals could significantly decrease population growth rates and size. One such factor is palm‐heart harvesting, which directly causes the death of immature and reproductive individuals. Indeed, palm‐heart is considered as the most important nontimber forest product exploited in the Atlantic Forest, and its harvesting has been identified as the main cause of reductions in the range of *E. edulis* in nonprotected areas (Silva‐Matos et al., 1999). Thus, to maintain viable populations, it may be crucial to minimize the death of immature and reproductive individuals, especially by controlling palm‐heart harvesting in *E. edulis*. Such controls are especially important in populations located at the upper altitudinal limits, which have been less subject to harvest so far, and thus can contribute to maintaining genetic diversity and regional persistence of this threatened species.

In conclusion, our results suggest that small‐scale environmental changes near the upper altitudinal range limit are sufficient to reduce some of the vital rates of *E. edulis*. This leads to lower abundance and thus contributes to setting the upper altitudinal limit for this threatened palm. We suggest that the upper altitudinal limit of this species is mainly the result of lower temperatures, since these may be the main factor reducing seedling recruitment and delaying the recruitment of reproductive individuals. Thus, our results highlight the potential importance of local environmental conditions in setting the altitudinal range limits of plant species, and also the need for demographic studies on small spatial scales to elucidate the factors that shape these range limits. In addition, we suggest that global warming may cause an upward shift in the altitudinal range of this tropical palm, since in future, the temperatures at higher altitudes will probably be similar to current temperatures at lower altitudes (Nogués‐Bravo, Araújo, Martinez‐Rica, & Errea, 2007). Future demographic studies should focus on the lower altitudinal limits of *E. edulis*, to evaluate whether these populations are likely to suffer altitudinal range contraction and become further endangered under climate change. This information would facilitate better management and conservation efforts for *E. edulis*, and potentially many other species, since the palm fruits of this species represent a key resource for the animal community (Galetti et al., 1999).

## CONFLICT OF INTEREST

None declared.

## AUTHOR'S CONTRIBUTIONS

All authors conceived and designed the study; A.C.S collected the field data; A.C.S conducted the analyses and wrote the paper with significant contributions from all other authors.

## DATA ACCESSIBILITY

Data will be deposited in the Dryad Digital Repository after acceptance for publication.
